# Healthcare Experiences and Expectations of Atheists, Deists and Agnostics in Türkiye: A Qualitative Study of Mental Well‐Being

**DOI:** 10.1111/jpm.70148

**Published:** 2026-05-22

**Authors:** Güven Soner, Emel Güven, Ercan Tunç

**Affiliations:** ^1^ Faculty of Health Sciences, Department of Public Health Nursing Ondokuz Mayıs University Samsun Türkiye; ^2^ Faculty of Health Sciences, Department of Psychiatric Nursing Ondokuz Mayıs University Samsun Türkiye

**Keywords:** health inequalities, inclusive healthcare, nursing care, patient experience, religiously unaffiliated individuals

## Abstract

**Introduction:**

Understanding how belief systems, or their absence, shape healthcare experiences is crucial for developing equitable and inclusive healthcare services.

**Aim:**

This study aimed to explore the healthcare experiences and expectations of atheists, deists and agnostics residing in Türkiye, with a focus on their mental well‐being.

**Methods:**

A qualitative study was conducted in Samsun, Türkiye, between June and August 2025. Purposive sampling was used and in‐depth, semi‐structured face‐to‐face interviews were conducted with 17 participants. Data were analysed using Colaizzi's phenomenological method and participants' direct quotations supported the identified themes and subthemes. The study adhered to the COREQ criteria for reporting qualitative research.

**Findings:**

Five main themes emerged: (1) Holistic Perception of Health, (2) Identity and Invisibility, (3) Dual Experiences in Healthcare Services, (4) Contextual Vulnerability in Mental Well‐Being and (5) Expectations for an Egalitarian and Professional System. Many participants avoided disclosing their non‐religious identity to healthcare professionals; those who did often encountered judgemental attitudes or unsolicited religious advice. Such experiences appear to contribute to decreased trust and, in some cases, avoidance of healthcare services.

**Conclusion:**

The findings of this study suggest that atheists, deists and agnostics may experience forms of invisibility within healthcare settings in this context, which can adversely affect their mental well‐being. Providing neutral, equitable and empathy‐based care, improving access to mental health services and training healthcare professionals, including nurses and physicians, to be sensitive to diverse belief systems are critical steps towards developing inclusive and human‐centred health policies in Türkiye.

**Relevance Statement:**

This study is relevant to psychiatric mental health nursing as it addresses the overlooked mental health experiences of atheists, deists and agnostics, a group often rendered invisible within healthcare systems. By exploring how stigma, exclusion and religious bias influence their psychological well‐being and trust in care providers, the study contributes to understanding the social determinants of mental health. The findings emphasise the need for nurses to deliver non‐judgemental, empathy‐based and culturally competent care that acknowledges diverse belief systems. Integrating such inclusive approaches into mental health nursing practice can enhance therapeutic communication, trust and overall psychological safety for all patients.

**Implications for Practice:**

Healthcare professionals often overlook the needs of non‐religious individuals, resulting in reduced trust, avoidance of care and weakened mental well‐being. This study highlights the importance of empathy‐based, neutral and inclusive care practices. Training nurses and other healthcare professionals to recognise and respect diverse belief systems will enhance communication, strengthen patient trust and contribute to equitable, human‐centred healthcare delivery.

## Summary

- What is already known?
  ○ Religious beliefs influence patients' experiences and expectations within healthcare systems.
  ○ Non‐religious individuals (atheists, deists, agnostics) often face invisibility or bias in healthcare settings globally.
  ○ Experiences of judgement or unsolicited religious advice in healthcare can negatively impact mental well‐being and service utilisation.
- What does this paper add to existing knowledge?
  ○ Provides the first qualitative insights into how atheists, deists and agnostics living in Türkiye experience the healthcare system.
  ○ Highlights that concealment of non‐religious identity is common, leading to reduced trust, heightened vigilance and occasional avoidance of care.
  ○ Emphasises the need for neutral, inclusive and empathy‐based healthcare practices, with targeted training for healthcare professionals to respect diverse belief systems.
- What are the implications for practice?
  ○ Integrate diversity and inclusion training into nursing and medical education to raise awareness about the experiences and needs of non‐religious individuals, promoting empathy‐based and non‐judgemental communication.
  ○ Develop institutional policies and guidelines that emphasise neutrality and respect for all belief systems, including non‐belief, to ensure equitable and human‐centred healthcare delivery.
  ○ Encourage reflective practice and supervision among healthcare professionals to identify and mitigate personal biases, fostering trust, psychological safety and improved mental well‐being for all patients.

## 1 Introduction

Delivering healthcare services in an effective, equitable and inclusive manner requires careful consideration of individuals' social, cultural and identity based diversity (Abbott et al. 2022; McCaffree 2019). Within this context, individuals' religious beliefs, or the absence of them, can play a significant role in shaping access to healthcare, satisfaction with services and overall health experiences (Hayward et al. 2016; Walker et al. 2021). Atheists, deists and agnostics may become invisible within the healthcare system due to the social stigma and exclusion associated with holding a non‐religious identity. This invisibility may directly or indirectly influence their ability to benefit from healthcare services (Speed 2022).

Research has shown that atheists, deists and agnostics tend to be at a disadvantage in terms of mental well‐being, social support and health behaviours (Abbott et al. 2022; Brewster et al. 2021; Hayward et al. 2016). Therefore, an in‐depth exploration of this group's experiences with the healthcare system is essential for identifying implicit or explicit biases in healthcare delivery and developing more inclusive health policies (Gad et al. 2022). Existing studies suggest that atheists, deists and agnostics may encounter prejudice from healthcare professionals based on religious orientation, which can negatively impact their psychological well‐being (Brewster et al. 2021; Cragun et al. 2012; Karim and Saroglou 2024).

Mental or psychological well‐being is a multidimensional concept encompassing life satisfaction, psychological resilience, subjective happiness and a sense of meaning in life (Baldwin et al. 2021; Karim and Saroglou 2024). Among the factors influencing well‐being, the social environment, sense of belonging and perceived social acceptance play a key role (Brewster et al. 2021). Studies have reported that experiences of exclusion and marginalisation faced by atheists, deists and agnostics, particularly in societies where religious identity is considered a social norm, can weaken their mental well‐being (Speed 2022; Zavalsız and Şahin 2018).

In the context of Türkiye, research directly examining the healthcare experiences of atheists, deists and agnostics is almost nonexistent (Zavalsız and Şahin 2018). The majority of the Turkish population identifies as Muslim (Kardaş 2024). Considering that religious values play a defining role in both social and institutional structures in Türkiye, it remains unclear what challenges atheists, deists and agnostics face in accessing healthcare, how these experiences affect their mental health and what expectations they hold from the system. Limited studies suggest that atheists, deists and agnostics in Türkiye may have diverse healthcare experiences and expectations influenced by contextual factors such as family relations, religious practices and exposure to traumatic events (Zavalsız and Şahin 2018). This gap in the literature represents a critical issue for both the inclusivity of healthcare services and the right to equal access to care.

The healthcare experiences of non‐religious individuals are not only a local concern but also a matter of global health equity. In diverse societies where a specific belief system constitutes the social norm, those with non‐religious identities may face unique challenges in navigating healthcare environments (Hayward et al. 2016; Walker et al. 2021). Understanding these dynamics in Türkiye provides illustrative insights for nursing practice in other socioculturally similar contexts where secular voices might be underrepresented in health discourse. Therefore, this study aims to address this gap by providing a qualitative understanding of the healthcare experiences, expectations and evaluations of mental well‐being among atheists, deists and agnostics living in Türkiye.

This study is the first qualitative exploration of the healthcare experiences and mental well‐being of atheists, deists and agnostics living in Türkiye. While previous research has primarily focused on the influence of religious beliefs on health, the perspectives of non‐religious individuals have remained largely invisible in the healthcare literature. By foregrounding their voices, this study identifies how religious bias, invisibility and lack of neutrality within healthcare interactions can undermine trust and psychological well‐being. The findings fill a significant gap in understanding inclusive and equitable healthcare practices from a nursing and mental health perspective.

Within the nursing discipline, humanistic and caring‐based approaches provide a valuable lens for understanding patient experiences. In particular, Watson's Theory of Human Caring (Watson 2008) emphasises empathy, moral commitment and respect for human dignity, principles that align closely with the present study's focus on inclusive and non‐judgemental healthcare experiences.

### 1.1 Aim

This study aims to explore in depth the healthcare experiences and expectations of atheists, deists and agnostics living in Türkiye within the context of mental well‐being.

### 1.2 Research Questions

This study addressed the following research questions:

1. What are the healthcare experiences of atheists, deists and agnostics living in Türkiye?
2. How do the beliefs of atheists, deists and agnostics living in Türkiye influence their healthcare experiences?
3. What are the expectations of atheists, deists and agnostics living in Türkiye regarding healthcare services?
4. What are the suggestions of atheists, deists and agnostics living in Türkiye for developing more inclusive healthcare services?

## 2 Methods

### 2.1 Research Design

One of the core functions of qualitative research is to reveal the challenges experienced by groups that are marginalised or rendered invisible within social structures (Delaunay et al. 2020). Accordingly, a qualitative research approach was adopted to comprehensively understand the experiences of atheist, deist and agnostic individuals living in Türkiye regarding healthcare services, as well as their expectations of these services, within the context of mental well‐being. This approach enables a multidimensional and contextual evaluation of participants' subjective experiences, aligning with the study's objectives.

The study was conducted between June and August 2025 in Samsun, Türkiye, through face‐to‐face interviews with participants. A descriptive phenomenological design, one of the qualitative research approaches aimed at in‐depth exploration of how individuals experience and make sense of specific phenomena, was employed (Sundler et al. 2019; Leigh‐Osroosh 2021). In this method, researchers analyse participants' accounts of their lived experiences as they are presented; during this process, data are directly transformed into meaningful statements without any interpretation or preconception (Penner and McClement 2008; Leigh‐Osroosh 2021). The research process was structured and reported in accordance with the Consolidated Criteria for Reporting Qualitative Research (COREQ) (Tong et al. 2007) (Table 1).

**TABLE 1:** COREQ checklist and explanations of the study.

	Item	Guide questions/description	Explanations
Research team and reflexivity	Personal characteristics		
	1. Interviewer/facilitator	Which author/s conducted the interview?	Principal investigator
	2. Credentials	What were the researcher's credentials?	All authors hold a PhD.
	3. Occupation	What was their occupation at the time of the study?	The principal investigator and second author were research assistants in the field of public health nursing, while the last author was a research assistant in the field of psychiatric nursing.
	4. Gender	Was the researcher male or female?	The principal investigator and the last author are male, while the second author is female.
	5. Experience and training	What experience or training did the researcher have?	The researchers have received training in qualitative research methods.
	Relationship with participants		
	6. Relationship established	Was a relationship established prior to study commencement?	No, a relationship was not established prior to the commencement of the study.
	7. Participant knowledge of the interviewer	What did the participants know about the researcher?	Before the interviews, the principal investigator informed the participants about his personal goals and reasons for conducting the research.
	8. Interviewer characteristics	What characteristics were reported about the interviewer?	The interviewer had a strong interest in the research topic due to prior experience in the field of public health nursing. No significant biases or assumptions were reported.
Study design	Theoretical framework		
	9. Methodological orientation and Theory	What methodological orientation was stated to underpin the study?	Phenomenology, with individual interviews
	Participant selection		
	10. Sampling	How were participants selected?	Snowball sampling
	11. Method of approach	How were participants approached?	Face‐to‐face
	12. Sample size	How many participants were in the study?	17 participants
	13. Non‐participation Setting	How many people refused to participate or dropped out?	None
	14. Setting of data collection	Where was the data collected?	The data was collected in cafes with accessibility features.
	15. Presence of nonparticipants	Was anyone else present besides the participants and researchers?	None
	16. Description of sample	What are the important characteristics of the sample?	The sample consisted of Atheists, Deists, and Agnostics living in Türkiye.
	Data collection		
	17. Interview guide	Were questions, prompts, and guides provided by the authors?	The researchers developed a semi‐structured interview form based on a review of the relevant literature. Afterwards, three experts checked.
	18. Repeat interviews	Were repeat interviews carried out? If yes, how many?	No, repeat interviews were not carried out.
	19. Audio/visual recording	Did the research use audio or visual recording to collect the data?	Audio recording
	20. Field notes	Were field notes made during and/or after the interview?	During the interviews, simultaneous handwritten notes were taken.
	21. Duration	What was the duration of the interviews?	The duration of the interviews ranged from 35 to 40 min.
	22. Data saturation	Was data saturation discussed?	Detailed information was given in the Methods section.
	23. Transcripts returned	Were transcripts returned to participants for comment and/or correction?	Transcripts were returned to participants.
Analysis and findings	Data analysis		
	24. Number of data coders	How many data coders coded the data?	All authors
	25. Description of the coding tree	Did the authors provide a description of the coding tree?	Codes, themes, and sub‐themes were identified.
	26. Derivation of themes	Were themes identified in advance or derived from the data?	Themes were derived from the relevant data.
	27. Software	What software, if applicable, was used to manage the data?	Demo version of MAXQDA 2020
	28. Participant checking	Did participants provide feedback on the findings?	Participants requested no changes.
	Reporting		
	29. Quotations presented	Were participant quotations presented to illustrate the themes/findings? Was each quotation identified?	Yes, participant quotations were presented to illustrate the themes/findings, and each quotation was identified by participant number or pseudonym.
	30. Data and findings consistent	Was there consistency between the data presented and the findings?	Yes
	31. Clarity of major themes	Were major themes clearly presented in the findings?	Yes
	32. Clarity of minor themes	Is there a description of diverse cases or a discussion of minor themes?	Yes

### 2.2 Theoretical Framework

According to Watson (2008), human caring emphasises empathy, moral commitment and respect for human dignity. This approach views nursing as both a moral and relational practice that values authentic connection and understanding between the nurse and the person receiving care. In line with this view, the present study interpreted participants' healthcare experiences through a compassionate and patient‐centred lens. This humanistic orientation provided a framework for exploring how empathy, inclusivity and professional neutrality influence the mental well‐being and trust of individuals with diverse belief systems.

### 2.3 Participants

The study was conducted with 17 individuals who self‐identified as Atheist, Deist, or Agnostic and were selected using a purposive sampling method. Participants were chosen from diverse age groups, genders and socioeconomic backgrounds. Inclusion criteria included being over 18 years old, self‐identifying as an Atheist, Deist, or Agnostic, residing in Türkiye, having received healthcare services at least once and voluntarily agreeing to participate in the study.

The inclusion of deists alongside atheists and agnostics was intentional to capture the experiences of individuals who hold non‐traditional or non‐institutional belief systems. In a Muslim‐majority context such as Türkiye, deists may share similar experiences of invisibility or bias in healthcare settings, making them a meaningful group for understanding the broader spectrum of non‐religious perspectives.

To reach participants, initial interviews were conducted with three individuals meeting these criteria within the researcher's network. Subsequently, these individuals referred other eligible participants to the researcher. When inviting participants to the interviews, the study's purpose and its potential contribution to the literature were clearly explained. In qualitative research, sample size is determined based on data saturation (Saunders et al. 2017). Accordingly, interviews in this study were conducted until data saturation was achieved.

### 2.4 Data Collection

Interviews were conducted face‐to‐face using a semi‐structured interview guide, arranged in an equal‐level seating format and employing an active listening approach. The lead researcher conducted all interviews. To ensure participants' privacy and sense of security, interviews were held in quiet cafés free from distractions, selected according to participants' preferences. Each interview environment was arranged to allow participants to express themselves comfortably.

During the interviews, an audio recording was used with participants' consent and simultaneous handwritten notes were taken. The lead researcher also observed and documented participants' non‐verbal communication, including gestures, facial expressions and body language. At the end of each interview, participants were asked to share their thoughts and suggestions regarding the study and their feedback on the process was recorded and taken into consideration.

#### 2.4.1 Semi‐Structured Interview Guide

Data were collected using a semi‐structured interview guide developed by the lead researcher based on a review of the relevant literature (Abbott et al. 2022; Gad et al. 2022; Hayward et al. 2016). The guide was reviewed by three experts, including a qualitative research specialist, a sociologist and a public health nursing expert and was revised in line with their feedback. It included 11 sociodemographic questions and nine questions exploring participants' experiences with and expectations of healthcare services. At the end of each interview, participants were also invited to add any additional comments or suggestions related to the study.

The semi‐structured interview guide collected information on participants' socio‐demographic characteristics (e.g., age, education level, occupation, frequency of healthcare service use) and included questions such as: ‘Have you had positive or negative experiences while receiving healthcare services? Could you share these experiences?’, ‘How does being an Atheist/Deist/Agnostic affect your mental well‐being when receiving healthcare?’ and ‘Have you ever shared your Atheist/Deist/Agnostic identity with healthcare professionals? How was it received?’ (Table 2).

**TABLE 2:** Interview questions.

What does ‘healthcare’ mean to you?
2 Have you had any positive or negative experiences while receiving healthcare services? Could you share these experiences?
3 How does being an atheist, deist, or agnostic affect your mental well‐being when accessing healthcare services?
4 Have you ever shared your atheist, deist, or agnostic identity with healthcare professionals? If so, how was it received?
5 Have there been situations where you felt that your atheist, deist, or agnostic identity influenced your access to or experience with healthcare services? Could you elaborate?
6 During times when you felt mentally unwell or were going through difficulties, how did the healthcare system support you?
7 Do you think the healthcare system in Türkiye contributes to your mental well‐being? Why or why not?
8 What are your expectations from the healthcare system in Türkiye?
9 In your opinion, what can be done to make healthcare services more inclusive and responsive?

### 2.5 Data Analysis

Audio recordings obtained from the interviews were transcribed by the researchers and transferred into Microsoft Word documents. To more accurately reflect participants' statements, behavioural cues observed during the interviews (e.g., smiling, long pauses, emotional fluctuations) were also noted in the transcripts.

Data analysis was conducted using Colaizzi's (1978) seven‐step phenomenological method. In the first step, the first two authors thoroughly read the transcripts to familiarise themselves with the data. In the second step, the texts were analysed line by line to identify meaningful statements, which were then labelled with corresponding codes. In the third step, the resulting codes were reviewed and organised into themes. In the fourth step, the main themes were elaborated and enriched with descriptive statements. In the fifth step, relationships between main and sub‐themes were evaluated and findings were considered within a holistic framework. In the sixth step, the themes were re‐examined to provide more precise and more in‐depth explanations of the essence of the phenomenon under study. In the seventh and final step, participant validation was conducted by randomly contacting three participants to obtain feedback on the findings. Following this process, the researchers reached a consensus on the main and sub‐themes.

The analysis process was conducted using a cyclical and dynamic approach, where each step informed the next. To ensure systematic coding, the demo version of MAXQDA 2020 (VERBI Software 2019), a software commonly used in qualitative data analysis, was utilised.

### 2.6 Trustworthiness and Rigour

The trustworthiness of the study was ensured and reported in accordance with the four core criteria proposed by Lincoln and Guba (1985): credibility, dependability, transferability and confirmability. For credibility, participants' statements were transcribed verbatim into Microsoft Word documents without any interpretation or intervention. During the interviews, the researcher occasionally repeated participants' statements to confirm understanding and ensure data accuracy.

To ensure dependability, all interviews were conducted by the same researcher using the same semi‐structured interview guide, enhancing the internal consistency of the data. Regarding transferability, the research context was described in detail and participants' demographic characteristics and selection criteria were comprehensively reported. Selecting participants from diverse age groups, genders and socioeconomic backgrounds through purposive sampling increased the potential applicability of the findings to similar contexts.

For confirmability, the generated codes and themes were reviewed by experts to support the reliability of the analyses. Additionally, transcripts were shared with participants via mobile messaging during the member‐checking process. None of the participants requested any changes, which reinforced confidence in the accuracy of the data.

To ensure rigour in the descriptive phenomenological approach, we employed bracketing by consciously setting aside our prior assumptions and experiences regarding non‐religious individuals' healthcare encounters. This allowed us to focus on participants' lived experiences as they were presented. The essence of experience was captured by identifying the core meanings of participants' narratives, highlighting shared patterns and unique perspectives without imposing pre‐existing interpretations. These practices ensured that the findings authentically reflect participants' experiences and maintain fidelity to the phenomenological framework.

### 2.7 Research Team and Reflexivity

The research team consisted of three academics working as doctoral‐level research assistants in a university nursing department. The first and third researchers are male, while the second researcher is female. The lead researcher specialises in public health nursing with a focus on healthcare services for underserved populations; the second researcher also works in public health nursing and the third researcher specialises in psychiatric nursing with an interest in developing interventions to address the psychosocial support needs of disadvantaged populations. All three researchers have received training in qualitative research methods and their collaboration allowed for a comprehensive examination of the topic from multiple disciplinary perspectives. The research team collectively values human diversity, respects differences and acknowledges a wide range of belief systems. Awareness of these positions allowed the researchers to actively reflect on potential biases and ensure that data collection and analysis remained neutral and participant‐centred.

Reflexivity was systematically applied to acknowledge and critically examine how the researchers' positions, beliefs and assumptions could shape the study. The lead researcher was aware that their professional background and prior experiences might influence interactions with participants and interpretations of their narratives. To mitigate this, all researchers engaged in regular self‐reflection and discussions, documenting observations in a research diary to monitor potential biases. Open‐ended questions and a neutral stance were employed during interviews to ensure participants could share their experiences freely and transcripts were shared with participants for member‐checking, allowing feedback to inform final interpretations. During data analysis, the team collaboratively reviewed codes and themes, discussing divergent perspectives to avoid subjective interpretations.

Additionally, the researchers conducted an impartial literature review to identify potential preconceptions about the healthcare experiences of atheist, deist and agnostic individuals and consciously worked to separate these from the analysis. Professional neutrality was maintained throughout data collection, ensuring no judgemental language was used and creating a safe environment for participants. These reflexivity practices strengthened the credibility of the findings and ensured that participants' perspectives were authentically represented, while making transparent the potential influences of the research team's positions and beliefs on the research process.

### 2.8 Ethical Considerations

Ethical approval for the study was obtained from the Social and Humanities Sciences Ethics Committee of Ondokuz Mayis University (Decision No. 2025‐669, dated 30 May 2025). This research was conducted in accordance with the Declaration of Helsinki and the ethical standards of the National Research Committee. To facilitate participants' access to the interview locations, the addresses were communicated to them via mobile messaging.

Before the study, participants were provided with detailed information regarding the study's purpose, content, process and principles of confidentiality and verbal consent was obtained. Participants were assured that their identities would remain confidential, that research data would be used solely by the researchers and that no information would be shared with third parties. Prior to recording the interviews, participants were informed that the audio recordings would be used exclusively for research purposes and would be deleted upon completion of the study. Both verbal and written informed consent were obtained from all participants prior to data collection. Recordings were securely stored on an encrypted hard drive and deleted after the transcription process was completed.

## 3 Findings

This section presents the sociodemographic characteristics of atheist, deist and agnostic participants residing in Türkiye, as well as the qualitative data obtained regarding their experiences within the context of healthcare services.

Table 3 displays the sociodemographic characteristics of the participants. A total of 17 participants took part in the study, comprising nine females and eight males, aged between 27 and 41 years. Participants self‐identified as atheists (*n* = 6), deists (*n* = 2), or agnostics (*n* = 9). Their occupations varied widely, including teachers, software developers, lecturers, psychologists, a lawyer, a translator, a yoga instructor and a technology specialist, reflecting a diverse professional background. Most participants described their perceived economic status as moderate, while a few reported low or high economic conditions. The frequency of healthcare utilisation also varied: several participants stated that they sought healthcare services once or twice a year, whereas others reported visiting every 3–6 months. This diversity in demographic and occupational characteristics provided a rich basis for exploring the experiences and expectations of non‐religious individuals regarding healthcare services in Türkiye.

**TABLE 3:** Characteristics of participants.

Participants ID	Age^a^	Gender	Self‐identified belief	Occupation	Perceived economic status	Healthcare utilisation frequency
P1	32	Male	Agnostic	Graphic designer	Moderate	1–2 times a year
P2	40	Male	Deist	Technology specialist	Low	1–2 times a year
P3	41	Male	Deist	Civil servant	Moderate	Every 3 to 6 months
P4	30	Male	Agnostic	Teacher	Moderate	Every 3 to 6 months
P5	28	Male	Atheist	Software developer	High	1–2 times a year
P6	34	Female	Agnostic	Lecturer	Moderate	Every 3 to 6 months
P7	29	Female	Agnostic	Psychologist	Moderate	Every 3 to 6 months
P8	35	Female	Atheist	Teacher	Moderate	1–2 times a year
P9	29	Female	Agnostic	Teacher	Moderate	Every 3 to 6 months
P10	27	Female	Atheist	Psychologist	Low	Every 3 to 6 months
P11	31	Male	Atheist	Software developer	Moderate	Every 3 to 6 months
P12	27	Female	Atheist	Yoga instructor	Low	1–2 times a year
P13	38	Male	Agnostic	Lecturer	Moderate	1–2 times a year
P14	27	Female	Agnostic	Lawyer	High	1–2 times a year
P15	27	Male	Atheist	Software developer	Moderate	1–2 times a year
P16	27	Female	Agnostic	Editor	Moderate	1–2 times a year
P17	27	Female	Agnostic	Translator	Low	1–2 times a year

^a^ The participants were 31.12 ± 4.81 years old.

**FIGURE 1:**
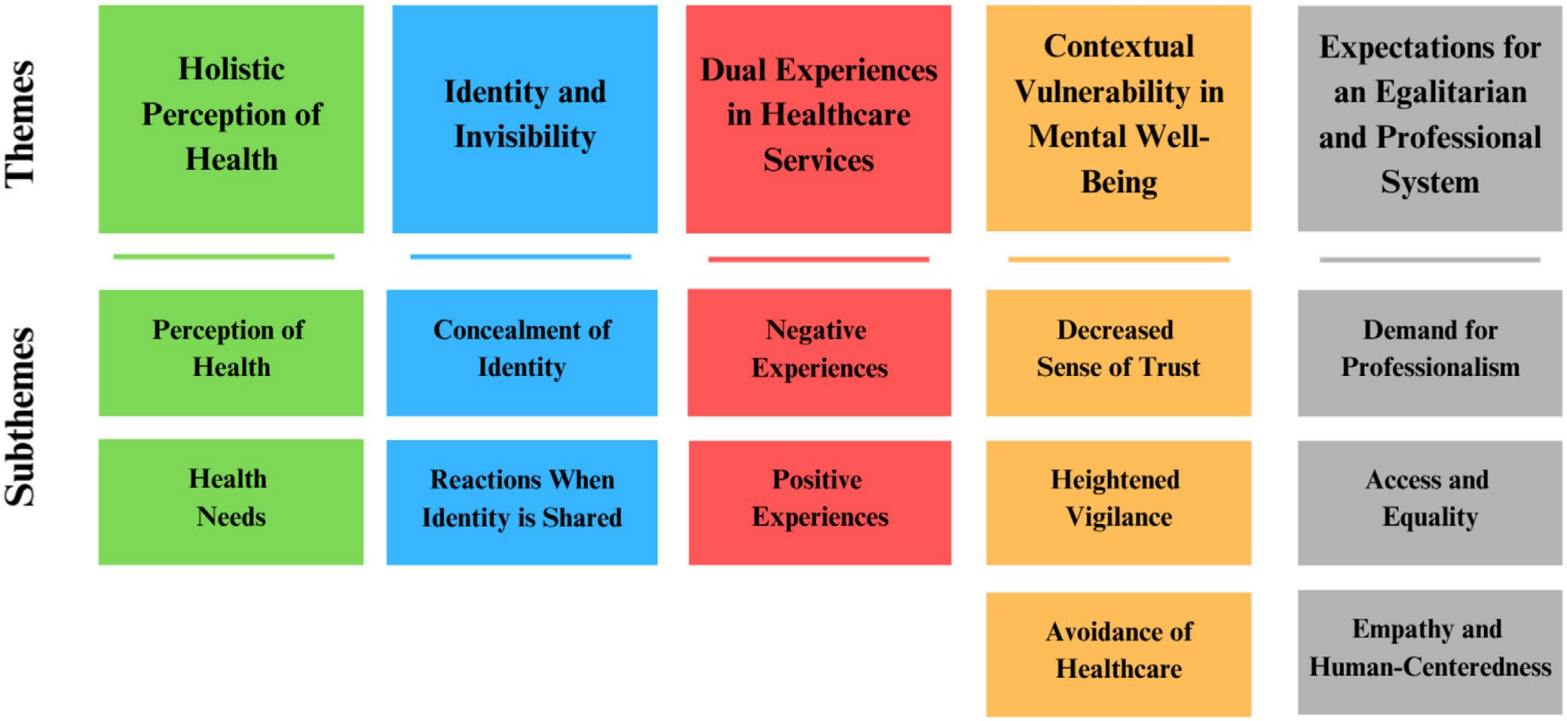
Themes and subthemes of the research.

Within the scope of the study, participants' experiences, as well as the meanings, were examined using a descriptive phenomenological approach guided by Colaizzi's method (Figure 1). The analysis resulted in the identification of five main themes and their corresponding subthemes. Quotations from the participants support each of the subthemes. The resulting themes, subthemes and codes are presented in Table 4. The analysis revealed five main themes reflecting participants' experiences and expectations regarding healthcare. Holistic Perception of Health indicated that participants perceived health as a holistic concept encompassing both physical and psychological dimensions and emphasised various health needs. Identity and Invisibility showed that many participants tended to conceal their non‐religious identity and when disclosed, they often encountered judgemental or distant attitudes. Dual Experiences in Healthcare Services reflected participants' coexistence of negative experiences, such as prejudice and exclusion, along with positive, professional and supportive encounters. Contextual vulnerability in mental well‐being was reflected in a decreased sense of trust, heightened vigilance and the avoidance of healthcare services due to fear or discomfort. Finally, expectations for an egalitarian and professional system reflected participants' emphasis on professionalism, equitable access to healthcare, empathy and human‐centredness in healthcare interactions.

**TABLE 4:** Themes, subthemes and codes of the research.

Themes	Subthemes	Codes	Quote
Holistic Perception of Health	Perception of Health	Physical + mental health, counselling, mental well‐being	*Health is not just the absence of illness; it is also being mentally balanced. (P2)*
	Health Needs	Psychological support, counselling, and accessibility issues	*Access to psychological support should be easy; it should not be limited only to those who can afford it. (P9)*
Identity and Invisibility	Concealment of Identity	Hiding atheist/deist/agnostic identity, ‘not necessary’ approach	*I don't tell the doctor about my identity because it's not related to health. (P4)*
	Reactions When Identity is Shared	Neutral/professional approach, negative reactions, and rare empathy	*Once I mentioned it, the doctor started giving religious advice, which made me uncomfortable. (P11)*
Dual Experiences in Healthcare Services	Negative Experiences	Religious references, prejudice, indifference, and access barriers	*The doctor told me to be patient and pray. Instead of support, I felt judged. (P8)*
	Positive Experiences	Empathy, explanatory communication, neutral attitude, and a trust relationship	*Some doctors explain everything very clearly, which provides reassurance. (P6)*
Contextual Vulnerability in Mental Well‐Being	Decreased Sense of Trust	Loss of trust in healthcare provider, concealing identity	*I didn't want to go to the same doctor again; my anxiety increased. (P13)*
	Heightened Vigilance	Experiencing stress before consultation, feeling anxious	*While waiting in the psychiatry department at a public hospital, I noticed the doctor treated clients very quickly and superficially. My interaction was ignored, and I felt some of my responses were met with ridicule. (P17)*
	Avoidance of Healthcare	Avoiding healthcare use, discontinuing treatment.	*Yes. Especially when seeking mental health support, some doctors' religiously framed approaches prevented me from speaking. Therefore, I didn't return to a few doctors. (P12)*
Expectations for an Egalitarian and Professional System	Demand for Professionalism	Service without religious references, unbiased approach	*I want them to focus on my illness, not my identity. (P12)*
	Access and Equality	Reducing financial burden, expanding psychological services	*Psychological support is expensive; the state should provide it. (P15)*
	Empathy and Human‐Centeredness	Listening, understanding, and a patient‐centred approach	*Even a little empathy can make a significant difference. (P10)*

### 3.1 Holistic Perception of Health

#### 3.1.1 Perception of Health

This sub‐theme reflects how participants conceptualised health. Participants described health not only as physical well‐being but as a holistic state encompassing mental and social dimensions. Importantly, their accounts predominantly emphasised psychological balance, personal well‐being and rational understanding, rather than spiritual or faith‐based interpretations. This orientation appears to be shaped by their non‐religious identities and provides an important context for understanding their expectations from healthcare services, particularly their preference for interactions grounded in scientific and non‐religious frameworks.

For me, health is not just the absence of illness. Feeling well both physically and mentally is important. For example, even if I do not have a headache, if my anxiety is high, I do not feel healthy. (P2)

Health for me includes being happy, feeling peaceful and being socially strong. Therefore, I find focusing solely on medication or physical treatment insufficient. (P5)

Health means that any health problem I experience gets resolved. Sometimes it also means being able to consult even when there is no problem. (P10)

#### 3.1.2 Health Needs

This sub‐theme highlights participants' expectations of healthcare services. Psychological support and counselling were frequently emphasised as essential components of care. Participants expressed a preference for accessible, evidence‐based and non‐judgemental services that address mental well‐being alongside physical health. These expectations appear to align with their broader non‐religious perspectives, where professional guidance and psychological support are prioritised over religious or spiritual approaches to coping and care.

Healthcare should include not only treatment but also counselling, preventive services and guidance. (P1)

Access to psychological support should be easy; it is a right for everyone, not just for those who can afford it. Today, finding a psychologist in a public hospital is almost impossible. (P9)

I think mental health services should be more accessible. People's minds, not just their bodies, should be treated. Currently, visiting private clinics is very expensive. (P12)

There should be an accessible, fair and non‐judgemental system. (P14)

### 3.2 Identity and Invisibility

#### 3.2.1 Concealment of Identity

This sub‐theme reflects situations in which participants chose not to disclose their Atheist, Deist, or Agnostic identities during healthcare encounters. Rather than representing a purely passive form of invisibility, participants' accounts suggest that non‐disclosure was often a deliberate and strategic choice. Many participants reported concealing their identities as a protective response to anticipated judgement, misunderstanding, or negative attitudes from healthcare professionals. This indicates that invisibility is, at least in part, actively produced by individuals navigating a sociocultural context in which non‐religious identities may be stigmatised.

I do not think the doctor needs to know that I am non‐religious because this information will not change the treatment, but it could expose me to judgement. Therefore, I do not disclose it unless asked; it is not relevant to health, but if I receive a negative reaction, my sense of trust is compromised. (P4)

My religious identity has been questioned since childhood. For this reason, I do not want to disclose it when visiting a doctor. In the past, when I told a nurse, she suggested prayers, which made me very uncomfortable. Now I never mention it. (P10)

I would not tell a psychiatrist at a public hospital that I am an atheist. There needs to be a certain level of trust. I do not think they could understand me. Trust has to be established first. In private hospitals, I usually select the doctor myself, so this is not an issue. (P11)

#### 3.2.2 Reactions When Identity Is Shared

This sub‐theme captures participants' experiences when their non‐religious identity was disclosed. While some participants encountered neutral and professional responses, others reported receiving unsolicited religious advice or encountering subtle forms of judgement. These experiences appear to reinforce participants' decisions to conceal their identities in future interactions, suggesting a cyclical process in which anticipated stigma shapes behaviour, which in turn sustains invisibility.

Especially when phrases like ‘pray’ or ‘faith is part of the treatment’ are used, I feel excluded or misunderstood. This can sometimes negatively affect my mood. (P2)

It is enough for healthcare professionals to be professional and not impose their religious beliefs on their work. Sometimes we see this in the news, which naturally worries me. For example, hearing expressions like ‘May God help you’, ‘God bless you,’ or fatalistic statements such as ‘there is a reason for this’ from healthcare providers is uncomfortable. Just being professional is sufficient; I do not want anything else. (P10)

Once, I explicitly told my psychiatrist that I am an atheist. During the conversation, he started talking about religious values and said, ‘Praying could also help.’ I was there to seek help, not to have my beliefs questioned. I felt diminished at that moment and did not want to return for another session. (P11)

### 3.3 Dual Experiences in Healthcare Services

#### 3.3.1 Negative Experiences

This sub‐theme reflects participants' negative experiences in healthcare settings. The most commonly reported issues were religiously framed statements, lack of attention and hurried care, all of which were described as undermining trust and continuity of care.

Last year, when I visited a family physician, after listening to my complaints, they said, ‘Be patient, pray.’ I expected support and solutions, but felt judged instead. As a result, I did not want to return to that doctor and interrupted my treatment. (P8)

I went to a neurologist for restless leg syndrome. The doctor was a religious person. At one point, the conversation turned to the brain's function and he said something like, 'There must be a divine power controlling our brains.' I found this unnecessary and it made me uncomfortable. This doctor broke my trust. (P11)

Before a surgery, a nurse kept giving me religious advice. I was already anxious and this increased my worry. At that moment, I only wanted the medical procedures to be explained and to be calmed down. (P13)

#### 3.3.2 Positive Experiences

This sub‐theme captures participants' positive experiences during healthcare encounters. Participants emphasised that healthcare professionals who were empathetic, communicative, neutral in personal beliefs and focused on clinical care fostered trust, safety and treatment adherence.

During my pregnancy, I told an obstetrician that I am a Deist. The doctor did not react at all and remained completely professional. They even said, ‘We are here for your health.’ This reassured me greatly and I felt safe. (P3)

Some doctors explain everything very clearly and provide a sense of security. For example, my surgeon explained each step before the operation, which reduced my anxiety. (P6)

I shared it with my psychologist. They responded with understanding. I had already chosen them after researching, so it was reassuring. (P11)

A nurse explained everything while performing procedures, which made me feel very relaxed. Knowing what was going to happen calmed me down. This kind of approach is very valuable. (P14)

### 3.4 Contextual Vulnerability in Mental Well‐Being

#### 3.4.1 Decreased Sense of Trust

Participants reported that biased or religiously framed attitudes from healthcare professionals weakened their trust in the healthcare system. This led to feelings of being misunderstood and undervalued. Loss of trust emerged as a key factor negatively influencing healthcare‐seeking behaviours.

Some healthcare professionals use religious statements unconsciously, which can make me uncomfortable. Especially regarding my lifestyle, I have sometimes felt a sense of moral judgement. This can occasionally weaken my trust in the treatment process. (P2)

I felt worthless and avoided going to the doctor for a while. As a result, my treatment was disrupted and I felt worse. (P4)

In mental health settings, sometimes being assumed to be a religious person led to recommendations of methods that were not suitable for me. This reduced my sense of trust. (P17)

#### 3.4.2 Heightened Vigilance

Some participants reported being constantly careful in healthcare settings due to concerns that their identity might be questioned. This state of ‘being on alert’ led them to moderate their communication and prevented full self‐expression, turning the healthcare experience into a psychologically tense and controlled process.

Even though it does not have a direct effect, I feel excluded in environments where religious references are frequent. This makes me constantly on alert while receiving healthcare, which creates stress. (P8)

Honestly, in some environments, I am constantly on guard because I feel the need to hide myself. Especially when receiving psychological support, fear that the professional might misunderstand me makes it difficult to be open and honest. (P9)

I did not want to go back to the same doctor; my anxiety increased. It felt as if they were more interested in my identity than my illness. (P13)

#### 3.4.3 Avoidance of Healthcare

Some participants who experienced negative encounters reported avoiding healthcare services to prevent similar situations from occurring. This avoidance behaviour was particularly evident in mental health services. Reluctance to seek treatment negatively impacted participants' physical and mental well‐being.

In a brief consultation with a family physician, I was told that ‘lack of faith could be the cause of depression’. This made me feel labelled and I did not choose that doctor again. (P1)

I did not experience direct discrimination, but sometimes, especially when seeking mental health support, the religiously oriented approaches of some centres discouraged me from using their services. (P5)

In a psychiatrist session, I brought up this issue. At first, they seemed to listen without judgement, but then said things like ‘faith keeps a person standing’. This pushed me away somewhat. (P6)

When I hear certain religious emphases, I withdraw. I cannot be completely open, which directly affects the quality of the service I receive. (P16)

### 3.5 Expectations for an Egalitarian and Professional System

#### 3.5.1 Demand for Professionalism

This sub‐theme reflects participants' expectation that healthcare services should be free from religious discourse and grounded in scientific and ethical principles. Professionalism was understood not only as technical competence but also as maintaining neutrality, clear boundaries and a focus on evidence‐based care. Even subtle religious expressions were perceived as potentially undermining patients' comfort and trust, particularly in vulnerable situations.

It is enough for healthcare professionals to be professional and not impose their religious beliefs on their work. Sometimes we see this in the news, which naturally worries me. For example, hearing expressions like ‘May God help you’, ‘God bless you’, or fatalistic statements such as ‘there is a reason for this’ from healthcare providers is uncomfortable. Just being professional is sufficient; I do not want anything else. (P10)

I want the focus to be on my illness, not my identity. It seems like a simple request, but it is often not respected. (P12)

Instead of religious advice, provide medical information. Just as I do not want to know the doctor's beliefs, they should not ask about mine. (P15)

#### 3.5.2 Access and Equality

This sub‐theme reflects participants' expectations for equitable access to healthcare services, regardless of socioeconomic status or belief orientation. They emphasised that services, particularly psychological support, should be accessible, affordable and free from financial barriers that limit care. Equal access was viewed as a fundamental requirement for a fair and inclusive healthcare system in which all individuals can receive appropriate and timely care.

Healthcare professionals should receive inclusivity and diversity training. The existence of non‐religious individuals and their sensitivities should be acknowledged. A healthcare environment where everyone feels safe should be created. (P3)

I want an egalitarian system where everyone can receive the same service. Currently, access to some services is minimal. (P10)

People should be treated equally as humans, regardless of their religious beliefs or lack thereof. I also want psychological support services to be more neutral and evidence‐based. (P14)

Psychological support is expensive; the state should provide it. The people who need it most are those without money. (P15)

#### 3.5.3 Empathy and Human‐Centeredness

This sub‐theme emphasises the importance of empathy, understanding and human‐centred communication in healthcare professionals' interactions with patients. Participants highlighted the need to be seen as whole individuals whose identities, values and lifestyles are acknowledged without judgement or differentiation based on belief systems. Empathetic engagement, even in simple forms, was described as a key factor that enhances trust, comfort and adherence to treatment.

More empathy, time and an approach that accepts the person's entire identity, including lifestyle, values and non‐religiosity. I also think mental health services should be more widespread and accessible. (P2)

I expect a more neutral, unbiased and human‐centred approach. It is important for me that services are provided without differentiation based on religion or non‐religiosity. (P3)

I want to be treated as a human. I am not just a file; when this is acknowledged, I adhere better to treatment. (P7)

Even a little empathy makes a big difference. When a nurse simply asks, ‘How are you?’ it is comforting. (P10)

The findings of this study have important implications for psychiatric‐mental health nursing practice. They emphasise the need for nurses to recognise belief diversity, including non‐religiosity, as a factor that affects mental well‐being and healthcare engagement. Incorporating training on empathy‐based and non‐judgemental communication into nursing education can help reduce stigma and strengthen therapeutic relationships. Fostering professional neutrality and awareness of unconscious bias can improve psychological safety, build patient trust and support equitable care for diverse populations with different beliefs.

## 4 Discussion

This study explored the healthcare experiences of atheists, deists and agnostics living in Türkiye, with a particular focus on mental well‐being. The findings are organised around three interconnected analytic insights. First, participants described a form of invisibility shaped not only by lack of recognition but also by the strategic concealment of non‐religious identities. Second, experiences of judgement and religiously framed interactions contributed to an erosion of trust, often accompanied by heightened vigilance and avoidance behaviours. Third, participants emphasised the importance of professional neutrality as a key condition for ethical, respectful and therapeutically effective care.

### 4.1 Invisibility as a Form of Perceived Exclusion

One of the most prominent findings of this study is the perceived invisibility of non‐religious identities within healthcare settings. However, participants' accounts suggest that this invisibility is not merely a passive condition, but is also actively produced through purposeful concealment of identity. Many participants reported choosing to withhold their beliefs, not because these identities were irrelevant to their care, but because disclosure was perceived as potentially unsafe within certain clinical contexts. In this sense, invisibility appears to function both as an absence of recognition and as a protective strategy shaped by anticipated stigma.

These findings are consistent with previous research indicating that non‐religious individuals may anticipate discrimination or exclusion in institutional settings, including healthcare (Abbott et al. 2022; Brewster et al. 2021). However, the present study extends this literature by demonstrating how concealment operates as an active communication strategy that shapes interpersonal dynamics and psychological safety within healthcare encounters.

Within this sample, religious identity was often implicitly assumed to be the default in healthcare interactions, which may have contributed to participants' sense of being positioned outside an unspoken ‘normative patient profile.’ In sociocultural contexts where religiosity is widely normalised, such assumptions may render non‐religious identities less visible or less legitimate in clinical encounters. This aligns with broader evidence suggesting that dominant cultural norms can influence whose identities are recognised and whose are marginalised in healthcare settings (Speed 2022; Cragun et al. 2012).

From a caring perspective, the need to conceal aspects of identity may constrain authentic presence and mutual openness, which are central to the development of trusting therapeutic relationships. When patients feel unable to express their values freely, the caring interaction may become partial or conditional, potentially weakening both relational depth and perceived safety (Watson 2008).

### 4.2 Erosion of Trust and the Emergence of Defensive Patient Behaviours

A second key insight concerns a perceived erosion of trust in healthcare professionals and systems among participants. Several participants described experiences such as perceived judgement, unsolicited religious advice, or subtle moral positioning, which contributed to feelings of being misunderstood or devalued. Rather than isolated incidents, these experiences were often described as cumulative, shaping expectations of future healthcare encounters.

These findings are consistent with previous literature demonstrating that perceived discrimination or value incongruence within healthcare interactions is associated with reduced trust, lower service utilisation and poorer mental well‐being (Hayward et al. 2016). In line with this, participants in the present study reported increased vigilance during healthcare encounters, including careful monitoring of communication and selective self‐disclosure. In some cases, this was accompanied by avoidance of healthcare services, particularly in mental health contexts.

Importantly, the findings suggest that trust is shaped not only by clinical competence but also by perceived relational and cultural safety. Participants' accounts indicate that even subtle expressions of personal belief by healthcare professionals may disrupt the therapeutic relationship, shifting the interaction towards a more defensive and self‐protective stance. From a human caring perspective, such disruptions may limit authentic engagement and weaken the relational foundation necessary for effective care (Watson 2008).

The coexistence of both positive and negative healthcare experiences within the same participant group suggests that care delivery is not uniform but rather context‐dependent and highly variable. This variability may be shaped by several interacting factors, including differences in communication skills, ethical sensitivity and cultural competence among healthcare professionals. Existing research highlights that healthcare providers' personal values and communication styles can either strengthen patients' trust or contribute to feelings of alienation (Hayward et al. 2016). In addition, structural factors such as workload, time constraints and institutional culture may further influence whether interactions are experienced as supportive or distressing.

### 4.3 Professional Neutrality as an Ethical and Therapeutic Imperative

The third analytic insight highlights that professional neutrality was perceived by participants as a key component of ethical and therapeutic care. Participants consistently expressed a preference for healthcare environments in which personal beliefs, whether religious or non‐religious, do not shape clinical decision‐making or interpersonal communication.

Within this study, neutrality was associated with fairness, respect and professionalism. Rather than reflecting passive detachment, neutrality was implicitly understood as an active practice requiring self‐awareness, reflexivity and the ability to maintain a patient‐centred approach. This interpretation aligns with broader discussions of culturally responsive care, which emphasise the importance of recognising and managing personal biases in clinical interactions.

The emphasis participants placed on neutral, evidence‐based care may also be understood in relation to their broader orientation towards health. As reflected in the findings, participants tended to conceptualise health in rational, psychological and scientific terms, rather than through spiritual or faith‐based frameworks. This aligns with previous research suggesting that non‐religious individuals often rely on secular and evidence‐based interpretations of health and well‐being (Speed 2022; Cragun et al. 2012). In this sense, expectations of neutrality may not only reflect a desire for fairness but also a need for epistemological alignment within healthcare encounters.

From a human caring lens, professional neutrality may be understood as an expression of moral commitment and respect for human dignity. Providing care that avoids the imposition of personal belief systems supports the development of authentic, trust‐based relationships and contributes to a sense of psychological safety. In this way, neutrality may enhance not only ethical practice but also the effectiveness of therapeutic interactions (Watson 2008).

### 4.4 Implications for Nursing Practice and Education

These findings have several implications for psychiatric mental health nursing and broader healthcare practice. First, they suggest that cultural competence frameworks may benefit from more explicit attention to non‐religious identities and worldviews. While existing models often focus on religious sensitivity, the present study indicates that sensitivity to non‐belief is also essential for inclusive care.

Second, nursing education may be strengthened by incorporating training on reflective practice, professional boundaries and awareness of how personal beliefs may influence clinical interactions. Developing structured training modules that address belief diversity, including non‐religious perspectives, may help healthcare professionals adopt more neutral and patient‐centred communication styles.

Finally, healthcare institutions may benefit from fostering environments that support reflective dialogue, supervision and ethical awareness regarding the role of personal beliefs in care delivery. Such efforts may contribute to building more inclusive, respectful and psychologically safe healthcare environments for diverse patient populations.

### 4.5 Strengths and Limitations

This study explored the healthcare experiences and expectations of atheist, deist and agnostic individuals living in Türkiye, with particular attention to participants' subjective perspectives. By foregrounding the voices of a relatively underrepresented group, the study provides insight into experiences of invisibility, perceived bias and diminished trust within healthcare settings. In doing so, it contributes to a growing body of research on inclusivity in healthcare and offers practical considerations for developing more equitable and respectful care practices.

Several considerations should also be acknowledged. The study was conducted within a specific sociocultural context. Participants were initially recruited through the researcher's network and subsequently via snowball sampling, which may have limited the diversity of perspectives represented in the sample. Future research could build on these findings by including more diverse participant groups across different regions and sociocultural contexts, incorporating healthcare providers' perspectives and conducting comparative studies across religious and secular settings. Additionally, quantitative or mixed‐methods follow‐up studies examining key constructs such as concealment, trust in healthcare providers and healthcare avoidance could further enhance understanding of how belief diversity influences healthcare experiences and interactions.

## 5 Conclusion

This study offers an in‐depth examination of the healthcare experiences and expectations of atheistic, deistic and agnostic individuals in Türkiye, with a particular focus on mental well‐being from a nursing perspective. The findings suggest that, within this sample, while some participants reported positive experiences with professional and supportive approaches from nurses and other healthcare providers, many also described experiences of perceived invisibility, judgement, or exposure to religiously framed attitudes. These experiences were reported by participants as being associated with increased anxiety and, in some cases, avoidance of healthcare services and diminished trust, which may have implications for their mental well‐being.

Participants in this study emphasised the importance of receiving healthcare in an impartial, equitable and professional manner. They highlighted the value of empathetic and patient‐centred communication from nurses and other healthcare professionals, regardless of personal religious beliefs. Key expectations included improved access to mental health services, the availability of psychological support for all individuals and training initiatives aimed at increasing healthcare professionals' awareness of diverse belief systems.

In conclusion, the findings of this study suggest that greater attention to belief diversity, including non‐religious worldviews, may be relevant for improving the inclusivity of healthcare services. From a nursing perspective, developing inclusive healthcare policies, strengthening cultural competence and enhancing awareness of belief diversity may contribute to more equitable and respectful care for patients with diverse belief systems and support a more patient‐centred healthcare environment. From the perspectives of public health nursing and psychiatric nursing, these findings underscore the importance of addressing belief diversity as part of holistic and person‐centred care. Both fields prioritise mental well‐being, social context and equity in healthcare access, highlighting the need for inclusive practices that recognise and respect diverse worldviews.

## Author Contributions

**Güven Soner:** conceptualization, methodology, data curation, formal analysis, writing – original draft preparation, writing – reviewing and editing. **Emel Güven:** conceptualization, formal analysis, writing – original draft preparation, writing – reviewing. **Ercan Tunç:** methodology, formal analysis, writing – original draft preparation, writing – reviewing.

## Funding

The authors have nothing to report.

## Ethics Statement

Prior to the study, ethical approval was obtained from the Social and Humanities Sciences Ethics Committee of Ondokuz Mayıs University (Decision No. 2025‐669, dated 30 May 2025).

## Conflicts of Interest

The authors declare no conflicts of interest.

## Data Availability

The data that support the findings of this study are available on request from the corresponding author. The data are not publicly available due to privacy or ethical restrictions.

## References

[jpm70148-bib-0001] Abbott, D. M. , D. Mollen , J. A. Boyles , and E. J. Anaya . 2022. “Hidden in Plain Sight: Working Class and Low‐Income Atheists.” Journal of Counseling Psychology 69, no. 1: 37–50. 10.1037/cou0000562. 34197150

[jpm70148-bib-0002] Baldwin, D. , J. Sinclair , and G. Simons . 2021. “What is Mental Wellbeing?” BJPsych Open 7, no. S1: S236. 10.1192/bjo.2021.631.

[jpm70148-bib-0003] Brewster, M. , B. Velez , J. Sawyer , W. Motulsky , A. Chan , and V. Kim . 2021. “Family Religiosity, Support, and Psychological Well‐Being for Sexual Minority Atheist Individuals.” Psychology of Religion and Spirituality 13, no. 3: 266–275. 10.1037/rel0000356.

[jpm70148-bib-0004] Colaizzi, P. F. 1978. “Psychological Research as the Phenomenologist Views It.” In Existential‐Phenomenological Alternatives for Psychology, edited by R. S. Valle and M. King , 48–71. Oxford University Press.

[jpm70148-bib-0005] Cragun, R. T. , B. Kosmin , A. Keysar , J. H. Hammer , and M. Nielsen . 2012. “On the Receiving End: Discrimination Toward the Non‐Religious in the United States.” Journal of Contemporary Religion 27, no. 1: 105–127. 10.1080/13537903.2012.642741.

[jpm70148-bib-0006] Delaunay, C. , A. Augusto , and M. Santos . 2020. “Invisible Vulnerabilities: Ethical, Practical and Methodological Dilemmas in Conducting Qualitative Research on the Interaction With IVF Embryos.” Societies 10, no. 1: 7. 10.3390/soc10010007.

[jpm70148-bib-0007] Gad, I. , X. C. Tan , S. Williams , et al. 2022. “The Religious and Spiritual Needs of Patients in the Hospital Setting Do Not Depend on Patient Level of Religious/Spiritual Observance and Should Be Initiated by Healthcare Providers.” Journal of Religion and Health 61, no. 2: 1120–1138. 10.1007/s10943-020-01103-7. 33128222

[jpm70148-bib-0009] Hayward, R. D. , N. Krause , G. Ironson , P. C. Hill , and R. Emmons . 2016. “Health and Well‐Being Among the Non‐Religious: Atheists, Agnostics, and no Preference Compared With Religious Group Members.” Journal of Religion and Health 55, no. 3: 1024–1037. 10.1007/s10943-015-0179-2. 26743877

[jpm70148-bib-0010] Kardaş, M. Ö. 2024. “The ‘Ascendance’ of Deism in Turkey: Context, Drivers and Debate.” Journal of Southeast European and Black Sea Studies 24, no. 3: 595–615. 10.1080/14683857.2023.2176197.

[jpm70148-bib-0011] Karim, M. , and V. Saroglou . 2024. “Agnostics' Well‐Being Compared to Believers and Atheists: A Study in Europe's Religious–Cultural Zones of Christian Heritage.” Religion 15, no. 12: 1502. 10.3390/rel15121502.

[jpm70148-bib-0012] Leigh‐Osroosh, K. 2021. “The Phenomenological House: A Metaphoric Framework for Descriptive Phenomenological Psychological Design and Analysis.” Qualitative Report 26, no. 6: 1817–1829. 10.46743/2160-3715/2021.4815.

[jpm70148-bib-0013] Lincoln, Y. S. , and E. G. Guba . 1985. Naturalistic Inquiry. Sage Publications.

[jpm70148-bib-0014] McCaffree, K. 2019. “Atheism, Social Networks and Health: A Review and Theoretical Model.” Secularism and Nonreligion 8, no. 9: 1–18. 10.5334/snr.101.

[jpm70148-bib-0023] Penner, J. L. , and S. E. McClement . 2008. “Using Phenomenology to Examine the Experiences of Family Caregivers of Patients with Advanced Head and Neck Cancer: Reflections of a Novice Researcher.” International Journal of Qualitative Methods 7, no. 2: 92–101. 10.1177/160940690800700206.

[jpm70148-bib-0015] Saunders, B. , J. Sim , T. Kingstone , et al. 2017. “Saturation in Qualitative Research: Exploring Its Conceptualization and Operationalization.” Quality & Quantity 52, no. 4: 1893–1907. 10.1007/s11135-017-0574-8. 29937585 PMC5993836

[jpm70148-bib-0016] Speed, D. 2022. “Godless in the Great White North: Assessing the Health of Canadian Atheists Using Data From the 2011/2012 Canadian Community Health Survey.” Journal of Religion and Health 61, no. 1: 415–432. 10.1007/s10943-020-01169-3. 33403601

[jpm70148-bib-0022] Sundler, A. J. , E. Lindberg , C. Nilsson , and L. Palmér . 2019. “Qualitative thematic analysis based on descriptive phenomenology.” Nursing Open 6, no. 3: 733–739. Portico. 10.1002/nop2.275. 31367394 PMC6650661

[jpm70148-bib-0017] Tong, A. , P. Sainsbury , and J. Craig . 2007. “Consolidated Criteria for Reporting Qualitative Research (COREQ): A 32‐Item Checklist for Interviews and Focus Groups.” International Journal for Quality in Health Care 19, no. 6: 349–357. 10.1093/intqhc/mzm042. 17872937

[jpm70148-bib-0018] VERBI Software . 2019. MAXQDA 2020 [Computer Software]. maxqda.com.

[jpm70148-bib-0019] Walker, M. H. , L. Drakeford , S. Stroope , J. O. Baker , and A. L. Smith . 2021. “Health Differences Between Religious and Secular Subgroups in the United States: Evidence From the General Social Survey.” Review of Religious Research 63, no. 1: 67–81. 10.1007/s13644-020-00430-1.

[jpm70148-bib-0020] Watson, J. 2008. Nursing: The Philosophy and Science of Caring. Rev. ed. University Press of Colorado.

[jpm70148-bib-0021] Zavalsız, Y. S. , and E. Şahin . 2018. “Ateist Ve Deistlerin Din Algısı: Üniversite Öğrencileri Üzerine Psiko‐Sosyolojik Bir Araştırma/Approachs of Atheists and Deists to Religion: A Psycho‐Sociological Research on University Students.” Journal of History, Culture and Art Research 7, no. 2: 567. 10.7596/taksad.v7i2.1425.

